# Factors associated with HIV testing and condom use in Mozambique: implications for programs

**DOI:** 10.1186/1742-4755-9-20

**Published:** 2012-09-05

**Authors:** Sohail Agha

**Affiliations:** 1 Population Services International, 1120 19th Street, NW, Suite 600, Washington, DC, 20036, USA

## Abstract

**Background:**

To identify predictors of HIV testing and condom use in Mozambique.

**Methods:**

Nationally representative survey data collected in Mozambique in 2009 was analyzed. Logistic regression analysis was used for two outcomes: HIV testing and condom use.

**Results:**

Women at a higher risk of HIV were less likely to be tested for HIV than women at a lower risk: compared to married women, HIV testing was lower among never married women (OR = 0.37, CI: 0.25-0.54); compared to women with one lifetime partner, HIV testing was lower among women with four or more lifetime partners (OR = 0.62, CI: 0.47-0.83). Large wealth differentials were observed: compared to the poorest women, HIV testing was higher among the wealthiest women (OR = 3.03, CI: 1.96-4.68). Perceived quality of health services was an important predictor of HIV testing: HIV testing was higher among women who rated health services as being of very good quality (OR = 2.12, CI: 1.49-3.00). Type of sexual partner was the strongest predictor of condom use: condom use was higher among men who reported last sex with a girlfriend (OR = 9.75, CI: 6.81-13.97) or a casual partner (OR = 11.05, CI: 7.21-16.94). Being tested for HIV during the last two years was the only programmatic variable that predicted condom use. Interestingly, being tested for HIV more than two years ago was not associated with condom use. Frequent mass media exposure was neither associated with HIV testing nor with condom use.

**Conclusions:**

The focus of HIV testing should shift from married women (routinely tested during antenatal care visits) to unmarried women and women with multiple sexual partners. Financial barriers to HIV testing appear to be substantial. Since HIV testing is done without a fee being charged, these barriers are presumably related to the cost of transportation to static health facilities. Mechanisms should be developed to cover the cost of transportation to health facilities. Substantially increasing community-based counseling is one way of reducing the cost of transportation. Men should be encouraged to test for HIV periodically.

## Background

Mozambique suffers one of the highest burdens of human immunodeficiency virus (HIV) in the world: about 11.5% of Mozambicans ages 15-49 are HIV positive [1]. Because of a broad range of socio-economic, cultural, historical factors that have left the country weak in its ability to respond to the epidemic, HIV prevalence and incidence rates show few signs of abatement [2]. The key strategies employed by the government of Mozambique for the prevention of HIV transmission include increasing the adoption of HIV counseling and testing and the promotion of condom use. Yet, there is a virtual absence of published studies that have identified factors associated with the adoption of HIV testing and condom use.

The national strategy for HIV testing has focused on pregnant women who attend antenatal clinics. Despite campaigns to encourage testing and to promote its advantages as a facilitator of access to treatment, the overall level of HIV testing remains low in Mozambique - 34% among women and 17% among men [1]. Moreover, it is not known whether persons at higher risk of HIV transmission are more likely to be tested than those at lower risk. Being tested for HIV may act as a driver of changes in behavior, although the results of studies which have looked at the effects of being tested on changes in sexual behavior are mixed. Some studies show that being tested for HIV results in increased unprotected sex [3], while others show that HIV testing is associated with higher condom use [4] or with reduced risky behavior among those HIV positive [5]. Some evidence suggests a positive effect of HIV testing on condom use in Mozambique: a study that compared a cohort of men and women who tested for HIV to a similar cohort who attended outpatient clinics but were not tested found that the increase in condom use was greater among those who were tested [6].

Condom use was first promoted in Mozambique during the early 1990s during HIV/AIDS awareness campaigns implemented by the Ministry of Health. Condom social marketing was initiated in 1994. While increases in condom use have been recorded in Mozambique, with condom use in last sex among never married women rising to 45% and among never-married men to 37%, there is concern that the initial strategy used to promote condoms for use with occasional partners or with commercial sex workers has limited the extent to which condoms are acceptable to young people - few young people identify with the stereotypical images of high-risk groups presented in the media [7]. The assumption underlying AIDS awareness efforts in Mozambique has been that unmarried people should not have sex and that the occasional sexual encounter that a married person or a person in a live-in-partnership has should be protected by the use of condoms [7]. As a result of this approach, the stigma associated with condom use is thought to have limited the extent to which unmarried women can negotiate condom use with a regular partner or the extent to which married women can negotiate condom use within marriage. One previous study which examined predictors of condom use among Mozambican men and women ages 15-24 found that correct assessment of personal risk of contracting HIV was positively associated with condom use [8].

This study examines predictors of HIV testing and condom use in Mozambique, two important strategies used to reduce HIV transmission in the general population. There is a virtual absence of studies that have investigated the predictors of HIV testing and condom use in Mozambique. A better understanding of drivers of the adoption of HIV testing and condom use will help determine approaches that should be pursued in order to reach those who remain at risk.

## Methods

### Study design

The data for this study comes from a nationally representative survey of Mozambique, “Inquerito Nacional de Prevalencia, Riscos Comportamentais e Informacao sobre HIV e SIDA em Mozambique” [1] for which fieldwork was conducted between June and October 2009. Conducted by the Instituto National de Estatistica of Mozambique and ICF Macro, the INSIDA 2009 is a multistage cluster sample survey representative of all provinces of Mozambique. At the first stage, 270 urban and rural ennumeration areas were selected from a list of 45,000 ennumeration areas defined during the 2007 census of Mozambique. At the second stage, a fixed number of households were selected in each cluster: 22 households were selected in each urban and 24 in each rural cluster. All women and men 15-64 who were permanent residents of the households or visitors who had spent the night at the household were eligible for the interview. For each household, a household questionnaire and individual questionnaires for men and women were used.

### Questionnaires

The instruments for the individual interviews were based on the instruments for the AIDS Indicator Surveys developed by MEASURE-DHS, the Nelson Mandela HIV/AIDS Behavioral Risk and Media Impact Survey conducted by the Human Sciences Research Council of South Africa and the 2003 Demographic and Health Survey conducted in Mozambique . The instrument for individual interviews collected data on socio-demographic characteristics, fertility, marriage and sexual activity and knowledge and perception of HIV/AIDS.

### Outcome variables

Two primary outcomes are examined in this study. To measure use of counseling and testing, all respondents to the survey were asked the following question: have you ever been tested for AIDS? In this study, the analysis of predictors of HIV testing is restricted to respondents who reported (ever) having had sexual intercourse in the past. To measure use of condoms during the last sexual intercourse, respondents who had had sex during the last 12 months were asked the following question: the last time that you had sexual relations did you use a condom?

### Independent variables

Geographic area and residence: these include residence in Southern (Inhambane, Gaza, Maputo province and Maputo city), Central (Sofala, Manica, Tete and Zambezia), or Northern (Nampula, Niassa and Cabo Delgado) Mozambique. Categorization in these three provinces is based on the geographic location of these provinces but is also broadly consistent with the level of HIV prevalence in the country, which is highest in the Southern provinces of Gaza (25%), Maputo province (20%) and Maputo City (17%) and lowest in the Northern provinces of Nampula (5%), Niassa (4%) and Cabo Delgado (9%).

Demographic and socio-economic variables: independent variables used for the analyses include age (in five year age groups), marital status (never married, currently married, formerly married), education (none, primary, secondary or higher) and household wealth (in quintiles). HIV prevalence is highest among Mozambican women 25-29 and men 35-39. Never married men and women have lower HIV prevalence than those currently married and both groups have lower prevalence than those formerly married (i.e. those divorced, separated or widowed). Education is associated with higher HIV prevalence for both men and women.

Mass media: exposure to messages about counseling and testing or campaigns about condom use can be important in motivating men and women to adopt HIV testing or condom use. An assessment conducted within the first two years of the introduction of condom social marketing in Mozambique, in 1996-97, showed that higher levels of exposure to *JeitO* condom advertising was associated with higher condom use in last sex with a non-regular partner – even after controlling for age, education, household wealth and frequency of radio listenership [9]. Variables measuring daily radio listenership and daily television viewership are used in this analysis.

Knowing someone who has died of AIDS: previous studies have shown that knowing someone who has died of AIDS can be a powerful predictor of behavior change [10]. A study in Mozambique also found that persons who knew someone with AIDS made changes in their behavior to protect themselves against HIV [11].

Knowing someone who is getting ARV treatment: knowing someone who is getting treated for HIV/AIDS is likely to make a person be receptive to learning about their HIV status because of the possibility of treatment.

Number of lifetime sexual partners: a person who judges their risk of HIV correctly is more likely to use a condom [8].

Rating of quality of health services: a higher perception of the quality of available health services is likely to motivate a person to utilize health services.

### Statistical analysis

Because of differences in economic status, decision-making power, and resulting social and economic vulnerability, men and women have different opportunities and choices regarding the adoption of services and behaviors. All analyses were stratified by gender, resulting in four main analyses: HIV testing among women, HIV testing among men, condom use among men, condom use among women. Cross tabulations were used to examine relationships between independent variables and outcomes. All independent variables were significantly associated with outcome variables at the bivariate level in one or more of the four main analyses. Formal tests of significance were conducted only at the multivariate level, in logistic regression analyses. This permitted us to take confounding factors such as region of country and residence in urban or rural areas into account. Adjusted odds ratios of HIV testing and condom use are presented for women and men.

## Results

### Predictors of HIV testing among sexually experienced women

Table 1 shows characteristics of sexually experienced women in the sample and predictors of HIV testing. Column 1 of Table 1 shows characteristics of the sample. Column 1 shows that 33% of women in the sample are from the North, 41% are from the Central region and 26% are from the South. Over two-thirds of women live in rural areas. Less than one-third of women are 15-24 years old. About 74% of women in the sample are currently married, 7% have never been married and 19% were married at some point in the past. The level of formal education is low: only 11% of Mozambican women have secondary or higher education. Mass media exposure is low: about 30% of women listen to the radio daily, while 14% watch television daily.

**Table 1:** Sample characteristics and predictors of HIV testing among sexually experienced women

	**(1)**	**(2)**	**(3)**
	**Sample characteristics % (n = 6,091)**	**% of women who have been tested (n = 6,091)**	**Adjusted odds of HIV test (n = 6,091)**
Region			
North	33.1	23.0	1.00
Central	40.7	36.6	1.84 (1.31 – 2.57)
South	26.2	50.5	2.04 (1.53 – 2.72)
Residence			
Rural	69.1	28.4	1.00
Urban	30.9	52.3	1.47 (1.12 – 1.93)
Age			
15-19	11.6	37.3	2.98 (2.19 – 4.06)
20-24	18.3	50.6	5.53 (4.31 – 7.11)
25-29	15.8	46.5	4.74 (3.75 – 6.00)
30-34	14.7	41.7	3.99 (3.08 – 5.15)
35-39	12.1	30.7	2.68 (1.99 – 3.62)
40 and older	27.5	18.1	1.00
Marital status			
Currently married	73.9	36.0	1.00
Never married	7.1	43.3	0.37 (0.25 – 0.54)
Formerly married	19.0	32.1	1.01 (0.81 – 1.23)
Education			
None	34.1	24.5	1.00
Primary	54.8	36.1	1.19 (0.95 – 1.51)
Secondary or higher	11.1	68.6	2.33 (1.69 - 3.22)
Wealth quintiles			
Fifth (poorest)	18.9	18.5	1.00
Fourth	19.2	23.9	1.30 (0.92 – 1.82)
Third	20.0	31.9	2.13 (1.50 – 3.03)
Second	20.8	41.8	2.52 (1.76 – 3.62)
First (least poor)	21.1	59.7	3.03 (1.96 – 4.68)
Radio listenership			
Listens less often	69.6	33.6	1.00
Listens daily	30.4	40.6	0.84 (0.69 – 1.02)
Television viewership			
Views less often	85.9	31.5	1.00
Views daily	14.1	61.4	1.22 (0.98 – 1.52)
Knows person who died of AIDS			
No	66.9	28.5	1.00
Yes	33.1	50.4	1.60 (1.35 – 1.89)
Knows someone getting ARVs			
No	81.2	30.6	1.00
Yes	18.8	58.0	1.66 (1.37 – 2.01)
Number of lifetime sexual partners			
One	41.0	37.8	1.00
Two	28.0	37.5	0.88 (0.72 – 1.08)
Three	17.3	33.6	0.73 (0.57 – 0.92)
Four or more	13.7	28.8	0.62 (0.47 – 0.83)
Rating of quality of health services			
Bad or very bad/do not know	8.3	26.4	1.00
Reasonable	31.9	34.1	1.40 (1.07 – 1.84)
Good	47.8	36.1	1.46 (1.12 – 1.90)
Very good	12.0	45.0	2.12 (1.49 – 3.00)
Total	100.0%	35.8%	
R-squared			18.6%

One-in-three women report having known a person who died of AIDS. One-in-five women know someone who is getting ARV treatment. About 14% of women report having had four or more sexual partners during their lifetime. Respondents’ ratings of the quality of health services in Mozambique show that only 12% consider the quality of health services to be very good.

Column 2 of Table 1 shows cross tabulations between independent variables and HIV testing. Overall, about 36% of Mozambican women ages 15-64 have been tested for HIV. HIV testing is highest in the South (50%) and lowest in the North (23%). A higher proportion of urban than rural women have been tested for HIV (52% vs. 28%). HIV testing is highest among women 20-24 and declines with age: 51% of women 20-24 have been tested for HIV, compared to 18% of women ages 40 and older. A higher proportion of never married women have been tested for HIV (43%) than currently married (36%) or formerly married (32%) women.

HIV testing increases with education: 24% of women with no education, 36% of women with primary education and 69% of women with secondary or higher education have been tested. HIV testing rises with household wealth: 18% of women from households in the fifth quintile (poorest), 32% from households in the third quintile and 60% from households in the first quintile (least poor) have been tested for HIV. A higher proportion of women who listen to the radio daily compared to women who do not have been tested for HIV (41% vs. 34%); HIV testing is nearly twice as high among women who watch television daily compared to women who do not (61% vs. 31%).

HIV testing is higher among women who know a person who has died of AIDS (50% versus 28%) and among women who know someone who is getting ARV treatment (58% versus 31%). A lower proportion of women with four or more lifetime sexual partners have been tested than women with one lifetime sexual partner (29% versus 38%). HIV testing increases with the perceived quality of health services: 26% of women who consider the quality of services to be poor have been tested for HIV, compared to 45% who consider the quality of services to be very good.

Column 3 of Table 1 presents odds of being tested for HIV from a logistic regression analysis. The likelihood of HIV testing is significantly higher in the South and Central regions compared to the North, even after adjusting for other variables. Urban residence is associated with a higher odds of HIV testing. Women under age 40 are more likely to be tested for HIV than women 40 and older.

Respondents who are never married are less likely to be tested for HIV than married respondents once income, education and other variables are adjusted for. In other words, the direction of the relationship between marital status and HIV testing changes after adjusting for other factors showing that never married women - who are at greater risk of HIV infection [12] - are less likely to get tested.

After adjusting for other factors, secondary or higher education is associated with a higher odds of HIV testing but primary education is not. Wealth is associated with a higher likelihood of HIV testing: women in the first (least poor), second and third quintiles are significantly more likely to be tested for HIV than women in the fifth (poorest) quintile. Surprisingly, after adjusting for other variables, there is no association between frequent listenership of radio or frequent viewership of television and being tested for HIV.

Knowing someone who has died of AIDS increases the likelihood of being tested for HIV, even after adjusting for other factors. Knowing someone who is getting ARV treatment is also associated with a higher odds of being tested. The higher the number of a woman’s lifetime sexual partners the lower her likelihood of being tested for HIV. Higher perceived quality of services is associated with a higher odds ratio of being tested for HIV even after other factors are adjusted for.

### Effect of income on counseling and testing among women and men

**Figure 1:**
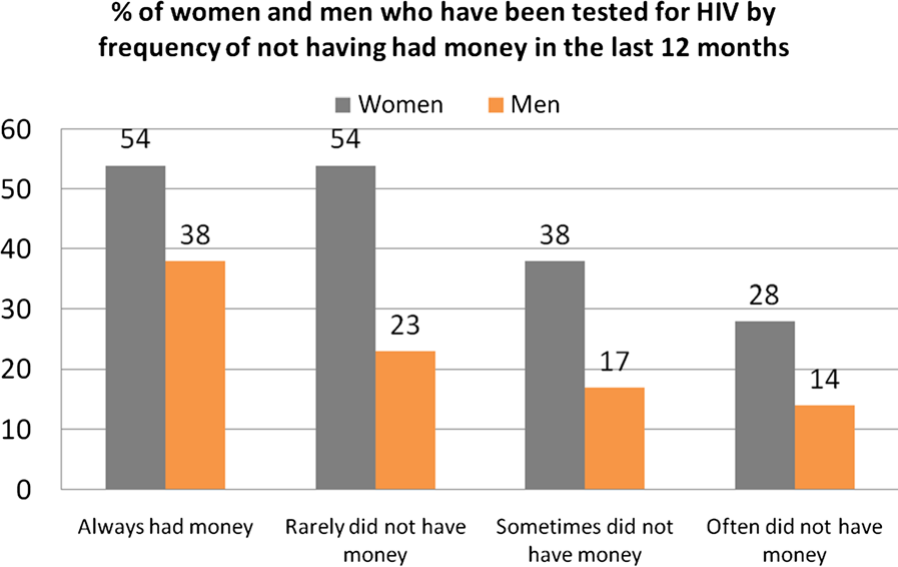
% of women and men who have been tested for HIV by frequency of not having had money in the last 12 months.

Figure 1 shows the level of HIV testing among Mozambican women and men by how often they did not have money in the last 12 months. There is a strong relationship between not having had money in the last 12 months and HIV testing among both women and men: about 28% of women who often did not have money in the last 12 months, 38% of women who sometimes had money in the last 12 months and 54% of women who always had money in the last 12 months were tested for HIV (p<0.001); about 14% of men who often did not have money, 23% of men who rarely did not have money and 38% of men who always had money were tested for HIV (p<0.001).

Odds ratios from logistic regression analysis conducted to identify predictors of HIV testing among men also showed powerful effects of household wealth on HIV testing: compared to men in the fifth (poorest) quintile, the odds of HIV testing was 1.74 (CI: 1.15-2.63) times higher among men in the third quintile, 2.46 (CI:1.63-3.72) times higher among men in the second quintile and 5.05 (CI: 3.33-7.64) times higher among men in the first quintile (results not shown).

### Predictors of condom use among men who have had sex in the last 12 months

Table 2 shows characteristics of men in the sample who have had sex in the last 12 months and predictors of condom use. Column 1 of Table 2 shows sample characteristics. In terms of their relationship with their last sexual partner, 67% of men who had sex in the last year had sex with their marital partner, 5% had sex with their live-in-partner, 21% had sex with their girlfriends and 7% had sex with a casual partner.

**Table 2:** Sample characteristics and predictors of condom use among Mozambican men who had sex in the last 12 months

	**(1)**	**(2)**	**(3)**
	**Sample characteristics % (n = 4,142)**	**% of men who used a condom during last sex (n = 4,142)**	**Adjusted odds of condom use (n = 4,142)**
Region			
North	36.5	6.3	1.00
Central	40.5	10.6	2.48 (1.53 – 4.01)
South	23.1	33.0	3.20 (2.14 – 4.79)
Residence			
Rural	65.7	6.0	1.00
Urban	34.3	29.8	1.46 (1.04 – 2.06)
Age			
15-19	12.3	34.4	2.54 (1.41 – 4.56)
20-24	15.1	28.5	2.71 (1.68 – 4.36)
25-29	15.2	14.8	2.29 (1.48 – 3.55)
30-34	14.1	10.8	1.99 (1.24 -3.18)
35-39	12.6	5.7	1.68 (0.93 – 3.04)
40 and older	30.6	3.8	1.00
Relationship with last sexual partner			
Marital	66.6	3.4	1.00
Live-in-partner	5.4	17.6	1.80 (0.70 – 4.61)
Girlfriend	21.3	38.0	9.75 (6.81 – 13.97)
Casual/other	6.7	43.2	11.05 (7.21 – 16.94)
Education			
None	12.4	3.1	1.00
Primary	64.0	7.5	1.34 (0.65 – 2.80)
Secondary or higher	23.5	38.4	3.19 (1.38 – 7.37)
Wealth quintiles			
Fifth (poorest)	16.6	2.5	1.00
Fourth	20.1	4.2	1.67 (0.78 – 3.58)
Third	20.6	5.8	1.73 (0.86 – 3.50)
Second	19.0	13.6	2.43 (1.23 – 4.80)
First (least poor)	23.7	38.6	3.99 (2.03 – 7.84)
Radio listenership			
Listens less often	56.5	11.8	1.00
Listens daily	43.5	17.3	0.99 (0.75 – 1.33)
Television viewership			
Views less often	81.4	8.8	1.00
Views daily	18.6	37.8	1.19 (0.91 – 1.55)
Knows person who died of AIDS			
No	62.2	11.4	1.00
Yes	37.8	18.7	1.07 (0.79 – 1.46)
Knows someone getting ARVs			
No	80.5	12.5	1.00
Yes	19.5	21.3	1.04 (0.78 – 1.38)
Number of lifetime sexual partners			
One	10.8	7.8	1.00
Two	17.2	13.1	2.10 (1.22 – 3.64)
Three	30.5	13.5	2.09 (1.24 -3.52)
Four or more	41.5	16.8	2.37 (1.41 – 4.00)
Have been tested for AIDS			
No	80.0	10.8	1.00
Yes, more than 2 years ago	5.3	19.5	1.34 (0.83 – 2.15)
Yes, within last 2 years	14.7	30.7	1.64 (1.14 -2.35)
Total	100.0%	14.2%	
R-squared			40.85%

Nearly one-quarter of men had secondary or higher education. About 43% of men listened to the radio daily, while 19% listened to the television daily. Nearly four-out-of-ten men knew someone who had died of AIDS and one-out-of-five knew someone who was getting ARV treatment. In terms of the number of lifetime sexual partners, about 30% of men had had three partners, while 41% had had four or more lifetime sexual partners. About 15% of men had been tested for HIV during the last two years while 5% had been tested more than two years ago.

Column 2 of Table 2 shows cross tabulations between independent variables and condom use in last sex. Overall, condom use in last sex among Mozambican men 15-64 was 14%. Use of condoms was much higher in the South (33%) than in Central (11%) or North (6%) regions. Condom use was higher in urban than in rural Mozambique (30% versus 6%). Use of condoms was higher among younger respondents and declined with age: 34% of 15-19 year olds, 15% of 25-29 year olds, and 4% of those 40 and older used a condom at last sex.

There was very substantial variation in condom use by type of sexual partner: condom use was higher with a girlfriend (38%) or casual partner (43%) than with a marital (3%) or live-in (18%) partner. Condom use also varied considerably by education and income: condom use was 3% among men with no formal education, 7% among men with primary schooling and 38% among men with secondary or higher education; condom use was 2% among men from households in the fifth (poorest) quintile, 6% among men from households in the third quintile and 39% among men in the first (least poor) quintile.

Before adjusting for other variables, condom use was higher among those with regular exposure to the mass media: a higher proportion of men who listened to the radio daily used a condom compared to men who did not (17% vs. 12%); a higher proportion of men who watched the television daily used a condom compared to men who did not (38% vs. 9%).

Use of condoms was higher among men who had known a person who had died of AIDS compared to men who did not (19% vs. 11%), and among those who knew someone who was getting ARV treatment (21% vs. 12%). Condom use increased with the number of lifetime sexual partners: 8% of men with one lifetime sexual partner used a condom during last sex and 17% of men with four or more lifetime sexual partners used a condom during last sex. Men who had been tested for HIV during the last two years reported higher condom use at last sex than men who were tested more than two years ago or men who had not been tested for HIV (31%, 19% and 11% respectively).

Column 3 of Table 2 shows the adjusted odds of condom use among men who had sex during the last 12 months. After adjusting for other factors, the odds of condom use in the Central and South regions of Mozambique remained significantly higher than in the North. Urban residence was associated with higher odds of condom use in last sex. Compared to men ages 40 and older, condom use was more likely among men ages 15-34. The likelihood of condom use was much higher with a girlfriend or a casual partner than with a marital partner. Secondary or higher education was associated with a higher likelihood of condom use. Men in the first (least poor) and second quintiles were significantly more likely to use a condom than men in the fifth (poorest) quintile. Having had more lifetime sexual partners was associated with a higher likelihood of condom use. HIV testing within the last two years increased the odds of condom use.

After adjusting for other variables, daily radio listenership and daily television viewership were not associated with condom use. Knowing a person who had died of AIDS or knowing someone who was getting ARV treatment were also not associated with condom use, once other variables were adjusted for.

## Discussion

After adjusting for other factors, never married women and women with a higher number of lifetime sexual partners are less likely to be tested for HIV. There is an urgent need to develop interventions that reach women at higher risk of HIV transmission in Mozambique.

Household wealth remained a powerful predictor of getting an HIV test, even after adjusting for education and a range of other variables. Large differentials in being tested were also observed for both women and men by a variable measuring how often the household did not have access to money during the last year. These findings suggest that the lack of financial resources at the household level is one of the largest barriers to obtaining an HIV test in Mozambique.

Distances to health facilities are large in Mozambique and transportation costs are a powerful deterrent to the utilization of health services. In 2009, with more than 90% of HIV tests being conducted in public sector facilities where services are provided at no charge, the cost of transportation to health facilities may have been prohibitive for many people. Community-based testing for HIV is being increasingly implemented in Mozambique and is likely to be very important in lowering the cost of transportation. However, the extent to which it is possible to reach a large segment of the Mozambican population through community-based HIV testing needs to be determined. At the same time, other approaches should be developed to lower the cost of transportation to static health facilities. Demand-side-financing approaches have proven to be a powerful tool to increase the utilization of health services within relatively short periods of time in a range of health areas [13]. Small-scale experiments suggest this strategy may be a powerful way of increasing the use of HIV testing among the poor [14]. Such an approach could be particularly important for men, who have less frequent contact with health services than women (the level of testing for HIV among Mozambican men is about half that of Mozambican women).

Women’s perceptions of quality of services are an important predictor of their getting tested for HIV. Clients are often unable to judge whether the technical quality of care provided is sufficiently high. Aspects of quality that are observed by clients such as waiting time, provider courtesy, availability of medicines often drive clients’ perceptions of quality and satisfaction with services [15-17]. The initial focus of improvements in service quality could be in areas which are directly experienced by clients and determine their perception of quality.

Residence in the North is associated with a disadvantage in terms of being tested for HIV even after adjusting for a range of observed variables, including urban residence, education, wealth and marital status. The provision of health services is weaker in the North than in the other regions, which may contribute to lower levels of service utilization. In addition, cultural and lifestyle factors are different in the North compared to other regions: about half the population of the North is Muslim compared to less than 10% of the population in the Central and South regions; the prevalence of male circumcision is higher than 90% in the North compared to around 50% in the South and 20% in the Central region.

The experience of knowing someone who had died of AIDS or knowing a person who was getting treated with ARVs were significant predictors of being tested, even after adjustment for other factors. In other words, the personal experience of observing AIDS-related illness or death are important drivers of being tested for HIV in Mozambique, while exposure to more impersonal communications messages are not. An earlier study documented how AIDS illness or death of a family member or friend motivated changes in sexual behavior [11]. Similar to the lack of impact of mass media exposure on HIV testing, no association between frequent mass media exposure and condom use was found after other factors were taken into account. There is little available in the published literature linking mass media campaigns implemented during the last decade with the adoption of condom use in Mozambique. A study conducted nearly fifteen years ago, within 18 months of the launch of the *Jeito* condom brand in Mozambique, showed that the social marketing campaign to promote *Jeito* had a significant, positive, impact on condom use in non-regular partnerships [9].

Findings from our study indicate that Mozambican men make the decision to use condoms based on the perceived risk of being infected by HIV from their sexual partner. Consistent with a perceived higher risk of HIV infection associated with having more sexual partners, condom use is higher for men who have a riskier sexual profile: the greater number of lifetime sexual partners the more likely a man is to use a condom in last sex, even after adjusting for type of last sexual partner and other variables.

There are significant differentials in condom use by wealth even after adjusting for education, type of last sexual partner and other variables. This suggests that the price of condoms or the costs associated with obtaining condoms (including price and transportation costs) remain a barrier to condom use. Ensuring widespread availability of condoms should remain a priority. This is particularly important in rural areas where poverty is much higher and the costs of transportation may be prohibitive. Only 15% of Mozambican men were tested for HIV in the two years before this study. Our findings suggest that ramping up the provision of HIV testing for men is probably the most viable programmatic strategy for getting substantial increases in condom use. Mechanisms to lower the cost of transportation to reach health facilities should be developed and community based counseling should be ramped up.

This survey was subject to many of the same limitations found in other cross sectional surveys. Specifically, the survey collected information on reported VCT use and reported condom use. This may be a cause of potential responder bias. Another potential limitation of this study is that it used condom use in last sex as an outcome whereas consistent condom use is more important for HIV transmission. Finally, no casual inferences should be drawn from a cross sectional study such as this.

## Competing interests

The author declares that there are no competing interests.

## Authors’ contributions

SA was responsible for the design of the study, the data analysis and for the write-up of the report.
